# Substrate for Thyroid Hormone Synthesis: Biochemistry, Evolution, and Physiology

**DOI:** 10.1096/fj.202600673R

**Published:** 2026-04-14

**Authors:** Crystal Young, Peter Arvan

**Affiliations:** ^1^ Division of Metabolism, Endocrinology & Diabetes University of Michigan Ann Arbor Michigan USA; ^2^ Department of Molecular & Integrative Physiology University of Michigan Ann Arbor Michigan USA

## Abstract

The use of iodotyrosines to control development and other biological processes predates the evolution of the thyroid gland. Endogenous thyroid hormone synthesis evolved with the ability to enzymatically iodinate protein secreted and directed for gastrointestinal proteolysis—and evolved further to specialize for iodination of protein entrapped extracellularly within closed follicles. The evolutionary appearance of the vertebrate thyroid gland with its follicular architecture correlates closely with the first appearance of thyroglobulin, which acts as the primary protein scaffold for thyroid hormone synthesis. Thyrocytes synthesize thyroglobulin in vast quantity, iodinating both hormonogenic and non‐hormonogenic tyrosines on the protein in the follicular lumen, where it serves as the body's supply of stored iodide (mono‐ and di‐iodotyrosine) as well as thyroid hormone (primarily T_4_, thyroxine). Endocytic ingestion of follicular thyroglobulin for delivery to lysosomes proteolytically liberates these residues for T_4_ release to the bloodstream as well as intrathyroidal iodide recycling. Humans bearing mutations impairing any steps leading to thyroglobulin iodination present with congenital hypothyroidism; nevertheless, untreated goitrous patients bearing bi‐allelic mutation in thyroglobulin can still make thyroid hormone. Even as thyrocytes in such patients grow to form a goiter, many thyrocytes die in the setting of persistent thyroidal endoplasmic reticulum (ER) stress. Recently, mice with genetic deletion of thyroglobulin have been found to also exhibit net goiter growth but also substantial thyroid cell death despite the complete absence of thyroidal ER stress. These findings suggest the possibility of a surprising back‐up mechanism in both patients and mouse models bearing bi‐allelic thyroglobulin mutation, that can link the iodination machinery for thyroid hormone synthesis to thyroid cell death.

## Graphical abstract

Two proposed sequence of events for thyroxine formation in patients expressing mutant thyroglobulin. (A) (1) TSH stimulation drives expression of misfolded TG. (2) Unremitting ER stress. (3) Stress‐induced cell death. (4) Dead thyrocytes release their contents. (5) The contents become iodinated. (6) Forming thyroxine. (B) (1) TSH‐stimulated proliferation. (2) Growth of thyrocytes into the follicle lumen. (3) Iodination of the exposed thyrocytes. (4) Cell death. (5) Iodination of dead cell contents. (6) Thyroxine formation.

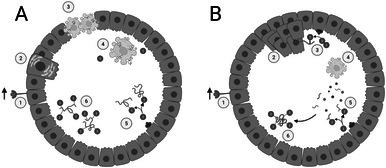

## 1 Overview of Functional and Hypofunctional Thyroid States

The thyroid gland plays a major role in many biological processes [1, 2], as the secretion of thyroid hormones thyroxine (T_4_) and triiodothyronine (T_3_) control phenotypes ranging from metamorphosis [3, 4, 5, 6] to growth and development, heart rate, and thermogenesis [7]. The most recognized action of thyroid hormone involves regulating the expression of genes controlling oxidative metabolism [8]. In humans, thyroid hormone affects virtually every organ system in the body including the cardiovascular system, central and autonomic nervous systems, bone, gastrointestinal system, and more [9].

Circulating thyroid hormone levels provide negative feedback to the hypothalamic–pituitary–thyroid (HPT) axis [10]. In the setting of a hypofunctional thyroid gland, hypothalamic sensing of low T_4_ (and T_3_) levels stimulates the release of thyrotropin‐releasing hormone (TRH) to drive the anterior pituitary gland to produce thyroid stimulating hormone (TSH). TSH positively acts on the thyroid gland to stimulate thyroidal iodide uptake and hydrogen peroxide (H_2_O_2_) generation, leading to increased production and secretion of thyroid hormone, as well as thyroid cell growth. Primary hypothyroidism is the clinical syndrome resulting from a hypofunctional thyroid gland that fails to generate adequate circulating thyroid hormone levels. Mild primary hypothyroidism may be only “subclinical,” with an elevated circulating TSH level sufficient to drive normal‐range levels of circulating T_4_. In more severe primary hypothyroidism, circulating thyroid hormone levels are sub‐normal despite the elevation of circulating TSH, and in such cases patients are likely to experience symptoms including cold intolerance, fatigue, dry skin, weight gain, hair thinning or hair loss, memory impairment, or difficulty with cognitive function [9]. The most common treatment for primary hypothyroidism is exogenous thyroid hormone, generally in the form of levothyroxine (T_4_) [11, 12]. The following short sections briefly discuss key gene products linked to thyroid hormonogenesis before considering the main topic of this review, which is the substrate protein upon which thyroid hormone is first formed.

## 2 Non‐TG Gene Products Involved in Normal Thyroid Hormonogenesis

At a microscopic level, the thyroid gland is organized into thyroid follicles—a spherical organization of cells that surround a central cavity [13]. Thyroid follicles are lined by a monolayer of thyroid follicular epithelial cells (also called thyrocytes) and the follicles serve as the basic unit of the gland for thyroid hormone synthesis [13]. Thyroid follicular cells are polarized, with the apical surface contacting the central follicular cavity, and the basolateral membrane and its underlying extracellular matrix leading to the bloodstream [13]. The interior cavity surrounded by follicular cells is known as the follicle lumen and is filled with proteinaceous “colloid,” which mostly consists of secreted thyroglobulin (TG), the precursor protein for thyroid hormone synthesis [13]. The follicles are surrounded by an extensive capillary bed and clustered together to form the highly vascular thyroid gland that is enclosed by a capsule [13]. Adjacent to thyroid follicular cells (which represent roughly half of all cells in the thyroid gland) are C‐cells (parafollicular cells) derived from the neural crest [14] as well as abundant fibroblasts, endothelial cells, and monocytes derived from the bloodstream [13].

For normal thyroid hormonogenesis, four biochemical components are essential: iodide (I^−^), hydrogen peroxide (H_2_O_2_), peroxidase activity, and TG (discussed below). Iodide absorbed from the gastrointestinal tract initially enters the thyroid by influx across the basolateral membrane of thyrocytes via the sodium‐iodide symporter (NIS; encoded by *SLC5A5*) [15], driven by the transmembrane Na^+^ gradient generated by the activity of the basolateral Na^+^/K^+^/‐ATPase [16]. The action of NIS allows for a roughly 30–60 fold increase in iodide concentration from plasma to thyrocyte cytosol [17, 18], from where it can efflux across the apical plasma membrane into the follicle lumen by various transporters including pendrin (PDS) [19], anoctamin 1 [20], and CIC5 [21].

Generation of H_2_O_2_ in thyrocytes involves the activity of dual‐function oxidase 2 (DUOX2) and DUOX1, both of which belong to the NADPH oxidase family. DUOX2 is the primary generator of H_2_O_2_ in the thyroid and is more highly expressed than DUOX1 [22, 23]. The DUOX maturation factors DUOXA2 and DUOXA1 facilitate the intracellular delivery of DUOX2 and DUOX1, respectively to the apical plasma membrane of thyrocytes and help to regulate the activity of these enzymes [24]. Given that H_2_O_2_ is cytotoxic to most cells, thyrocytes employ various defense mechanisms to provide resistance against these toxic effects [23, 25]. First, because the DUOX system is located at the apical surface of thyrocytes, much H_2_O_2_ resides extracellularly, protected by the highly glycosylated extracellular surface at the apical plasma membrane [26]. Second, effective antioxidant defenses help to limit the consequences of cytosolic H_2_O_2_; specifically, glutathione peroxidase (GPx), peroxiredoxins, and catalase may all contribute to breaking down physiological concentrations of H_2_O_2_ inside thyrocytes [27, 28, 29]. Finally, base‐excision repair and nucleotide excision repair exist as two major DNA repair mechanisms in thyrocytes that help to combat H_2_O_2_‐induced DNA damage [30].

**FIGURE 1:**
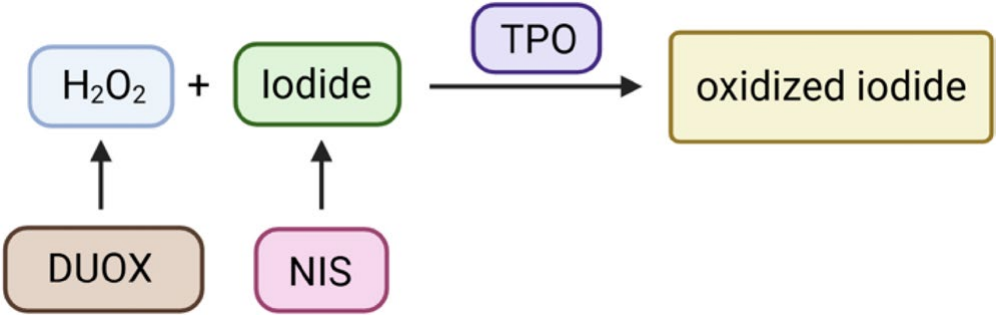
Formation of oxidized iodide. TPO utilizes H_2_O_2_ generated by the DUOX system and iodide transported to the follicular lumen (via NIS plus apical) iodide transporters to generate oxidized iodide, which becomes available to interact with protein tyrosine residues to produce 3‐iodotyrosine (MIT) and 3,5′‐di‐iodotyrosine (DIT).

Apical extracellular H_2_O_2_ and apical iodide become available to the catalytic domain of thyroid peroxidase (TPO), which uses its enzymatic activity for the generation of oxidized iodide (Figure 1) that is formed initially at the apical surface [31]. The oxidized iodide is highly reactive and triggers the iodination of tyrosine residues, yielding 3‐iodotyrosine (mono‐iodotyrosine, or MIT) and 3,5′‐di‐iodotyrosine (DIT) residues on secreted TG [32]. Many of these residues serve merely as the body's long‐term storage depot for iodide, but some of the earliest iodinated DIT residues within the TG molecule are used in coupling reactions to generate iodothyronines [32], discussed below.

## 3 The Thyroglobulin (TG) Gene Product in Normal Thyroid Hormonogenesis

**FIGURE 2:**
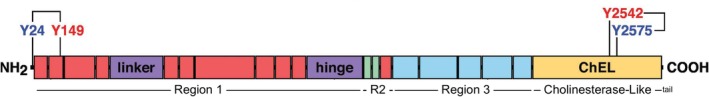
Sites of intramolecular diiodotyrosine coupling confirmed in (bovine) TG. This figure highlights the two primary thyroxine‐forming sites visualized by cryo‐EM in bovine TG (amino acid numbering from the bovine sequence), adapted from [37] (with permission). In contrast, coupling (not shown) of the antepenultimate iodotyrosines at the C‐terminal tail is thought to require an intermolecular interaction between TG monomers and favors de novo T_3_ formation.

TG is the main precursor protein for normal thyroid hormone synthesis in vertebrates. The human *TG* gene consists of a ~270 kb single‐copy gene localized on chromosome 8q24.22, with 48 exons ranging in size from 62 to 1101 nucleotides and introns that also vary in size [33, 34]. The presence of very high expression levels of TG protein is a defining feature of the thyroid, as it usually accounts for 75% of the total protein content of the mammalian thyroid gland [35]. Newly synthesized TG contains a ~19‐residue signal peptide that is removed upon successful delivery of the protein into the endoplasmic reticulum (ER) [36]. The remainder of TG is synthesized as a monomeric glycoprotein ~330 kDa in size and its primary sequence is divided into regions 1, 2, 3 and concludes with the Cholinesterase‐like region and a short unique C‐terminal tail sequence (Figure 2). The first three regions are designed as a chain of modules: four TG‐type 1 repeats, a linker segment, six additional type 1 repeats, a hinge segment, three TG‐type 2 repeats, a final type 1 repeat, and five TG‐type 3 repeats before the ChEL domain and a short unique tail of ~32 residues [34, 38, 39]. The repeating modules are cysteine‐rich, encoding intradomain disulfide bonds, with type 1 modules containing the central CWCV sequence and either six (type 1A) [40] or four cysteine residues (type 1B) [41]. Type 2 modules involve a short span of just 14–17 residues and contain two cysteines, and type 3 modules have eight (type 3A) or six cysteine residues (type 3B) [41].

The disulfide bonds in TG form primarily in the ER, assisted by endogenous oxidoreductases including ERp57 (assisted by glycan‐dependent calnexin and/or calreticulin chaperones), protein disulfide‐isomerase (PDI), ERp72, and P5/ERp46 [42, 43]. These oxidoreductases can facilitate disulfide bond formation in secretory proteins, and their isomerase activity can help to reshuffle non‐native disulfides to favor the new formation of proper disulfide bonds [42, 44]. The TG monomer folds slowly and eventually self‐associates noncovalently into a homodimer in the ER (~660 kDa) [45]. All of the above structural features of native TG have been essentially “visualized” within the electron density map of TG homodimers by cryo‐EM, supported by the work of several groups [37, 46, 47, 48]. Bearing close relationship to the regional primary sequence (Figure 2), five structural regions of each monomer in the 3D context were named: (i) the N‐terminal domain, (ii) “core,” (iii) “flap,” (iv) “arm,” and (v) C‐terminal domain [46]. In addition to homodimerization, after secretion some TG dimers can further oligomerize in the oxidizing environment of the follicular lumen [49, 50].

During trafficking in the intracellular secretory pathway, TG undergoes post‐translational modifications that can impact its hormonogenic potential. N‐linked glycosylation occurs during TG synthesis and is considered essential to TG protein folding and trafficking [51], immunoreactivity [51], as well as iodination and hormone synthesis [52, 53]. Glycan modifications occur during intracellular transport. Phosphorylation of TG occurs within carbohydrates as well as on serine and tyrosine, and may influence the efficiency of T_3_ formation within the TG molecule [54, 55]. These modifications, as well as sulfation of TG, which occurs as a late post‐translational modification in the trans‐Golgi network, appear to be regulated by the effects of TSH on thyrocytes [56]. Sites on TG favored for iodination and hormonogenesis are also favored for tyrosine sulfation, suggesting that tyrosine sulfation influences the TG sites that remain available for thyroid hormonogenesis [57].

**FIGURE 3:**
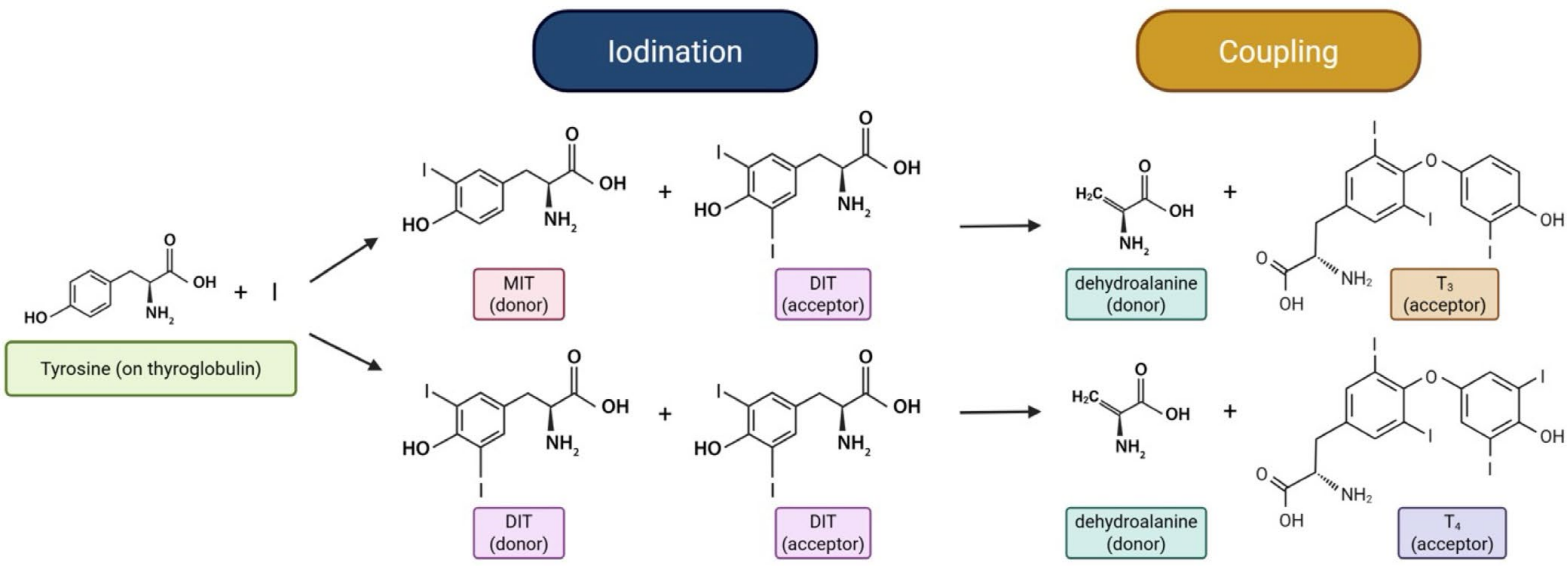
Coupling reaction to form T_4_ or T_3_. Thyroid peroxidase (TPO) catalyzes the iodination of specific tyrosine residues on thyroglobulin, forming mono‐iodotyrosine (MIT) and di‐iodotyrosine (DIT). Under oxidizing conditions, coupling of one DIT donor with one DIT acceptor forms dehydroalanine (at the donor site) and T_4_ (at the acceptor site, lower reaction in the figure); MIT donor coupling with DIT acceptor forms dehydroalanine and T_3_ (upper reaction in the figure). Upon coupling, both dehydroalanine and the newly‐formed iodothyronine remain within the polypeptide chain of TG itself, located at a few distinct, preferred hormonogenic sites (see Figures 2 and 4).

**FIGURE 4:**
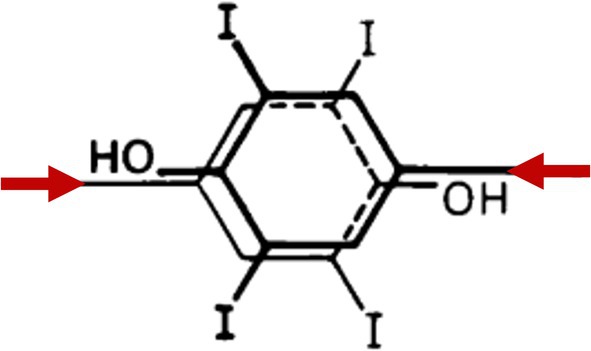
For coupling, two DIT residues align in an anti‐parallel orientation to avoid strain in the parental polypeptide chain. Adapted from [59] (with permission).

**FIGURE 5:**
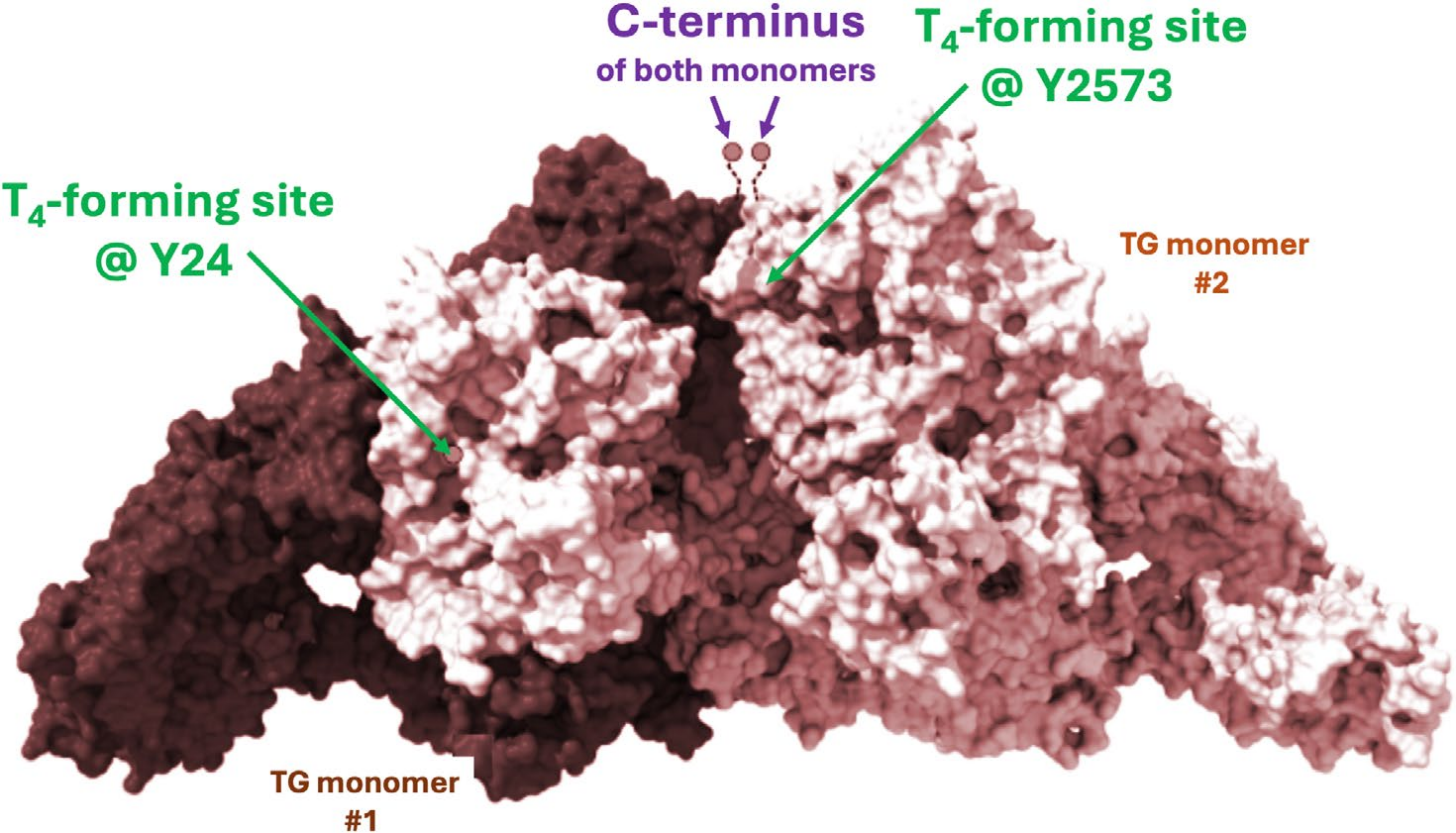
The three most used sites for thyroid hormone formation mapped onto the TG surface. The cryo‐EM model of human TG homodimer is adapted from [47] (with permission). The antepenultimate iodotyrosines of TG are used preferentially for T_3_ synthesis via a coupling reaction between the C‐terminal tails of each monomer within the homodimer.

Iodination of tyrosine residues on TG is the most important post‐translational modification in the thyroid hormonogenesis pathway. Initial formation of MIT and DIT occurs at specific sites within TG (discussed below), which are thought to be favored based on tyrosine residue exposure at the surface of the TG molecule [58]. Crucially, based on TG tertiary and quaternary structure, at the time of iodination, selective DIT pairs undergo a coupling reaction (Figure 3). The coupling of a DIT donor residue to a DIT acceptor residue will form dehydroalanine (at the donor site) and T_4_ (at the acceptor site) within the TG polypeptide backbone. Similarly, coupling of a MIT donor and DIT acceptor will form dehydroalanine (donor site) plus T_3_ (acceptor site) [32] (Figure 3). This reaction competes with initial iodination reactions, so coupling to make T_3_ can potentially occur before a donor MIT can be further iodinated to DIT [59]. In addition, after T_3_ formation through MIT‐DIT coupling, T_3_ cannot be further iodinated to be converted into T_4_ [59]. Therefore, all T_4_ synthesis in TG occurs through DIT‐DIT coupling [59]. Although non‐hormonogenic and not necessarily chemically favored, it is also possible to have some MIT‐MIT coupling to form T_2_, as well as DIT (donor) coupling to MIT (acceptor) to form reverse T_3_ [60]. The coupling reaction requires that the polypeptide backbone provides proper orientation of the phenolic rings of the donor and acceptor residues (Figure 4), pointing in opposite directions [59]. Evidently, these features are only found at limited sites within the 3D structure of the TG molecule (Figure 5).

The pH of the (murine) thyroid follicle lumen has been measured at 7.58 [61], and studies have shown that hormonogenic coupling after peroxidase activity is favored by alkaline pH (most favored at pH 8) [62]. As a result of iodination and coupling, under conditions of normal iodide supply, thyroidal TG contains on average 2.5 residues of T_4_ and 0.7 residues of T_3_ per TG dimer (as well as 4.5 residues of DIT and a further 5 residues of MIT) [63]. Overall, the process of synthesis and release of thyroid hormone from TG has been termed “first come, first served,” signifying preferential iodination and thyroid hormone formation on the most recently‐secreted TG molecules that reside in close proximity to the apical plasma membrane where oxidized iodide is first formed [64, 65].

A large fraction of iodinated TG remains in the follicular lumen for long‐term storage and is available for re‐internalization into thyrocytes when stimulated by TSH, via endocytosis [65, 66]. This provides a pool of pre‐formed thyroid hormone available to meet the body's needs in the event that subsequent iodide deficiency is encountered [67]. Upon TSH stimulation, endocytic internalization of TG delivers the iodoprotein to lysosomes where the polypeptide backbone is proteolyzed to liberate thyroid hormones that efflux from lysosomes to the cytosol. Thyroxine can then be transported via the basolateral plasma membrane to capillaries for release to the bloodstream [68]. Under stimulated conditions, some chemical proteolysis [63] and enzymatic proteolysis may begin even in the follicle lumen and early endocytic pathway, allowing for some initial cleavages in the TG molecule prior to its arrival in lysosomes for complete degradation [69, 70, 71, 72, 73]. Many distinct lysosomal proteases contribute to the lysosomal degradation of TG for liberation of thyroid hormones [70, 71, 73, 74, 75], and the rate of TG transfer to lysosomes correlates with hormone production [76, 77]. Lysosomal degradation of TG also yields a large supply of free MIT and DIT, which upon release to the cytosol is efficiently deiodinated by iodotyrosine dehalogenase 1 (IYD1) [78, 79, 80] to allow for intrathyroidal iodide recycling [78] that is essential for continued thyroid hormone synthesis in the setting of iodide deficiency.

The monocarboxylate transporter 8 (MCT8), which is expressed in a variety of cell types, can facilitate the efflux of cytosolic iodothyronines from thyrocytes to the bloodstream, as well as facilitating thyroid hormone influx into various cells and tissues across the body [68]. In addition, L‐type amino acid transporters LAT1 and/or LAT2 may function as secondary hormone transporters [81, 82, 83]. Beyond this, some intact TG may enter the bloodstream directly by secretion of newly‐synthesized TG that is misdirected to the basolateral side of the epithelial monolayer, or by leakage from disrupted follicles, or by transcytosis from the follicular lumen to the basolateral surface of thyrocytes [64, 65, 84, 85]. The net result of TG synthesis, iodination, coupling, endocytosis, proteolysis, and thyroid hormone transporter function is that the totality of the body's endogenous supply of T_4_ comes from de novo thyroid hormone biosynthesis in the thyroid gland [68].

## 4 Sites of Hormonogenesis on Thyroglobulin

Overall, the 3D structure of TG appears very complex and intertangled, revolving around a central ChEL dimer that interacts with different regions of the “arm” and “core” of one chain, and with the N‐terminal domain of the other chain [37, 46, 47, 48]. In each monomer, the N‐terminal domain connects to the “core” (which itself contains two separated triplets of type‐1 repeats) via a linker that crosses the central dimer interface and is partially flexible. Expression of truncated, recombinant TG suggests that this N‐terminal domain is the most difficult region of the protein to fold [86]. Many of the disulfide bonds of TG have been visualized, and no intersubunit disulfide bonds have been found, which is consistent with classic observations that TG is a noncovalent dimer [46, 87], with an extensive dimer interface (29 350 Å^2^) [46].

Within this structure, three main, conserved, hormonogenic acceptor sites (along with minor, less‐conserved sites) have been identified in the nearly 2750‐residue human TG protein (Figure 5). The most important T_4_‐forming site in vertebrates occurs at Y24 (“acceptor Site A”), which is the 5th residue after removal of the N‐terminal signal peptide (Figures 2 and 5) [88]. In most species, this acceptor site accounts for up to half of all T_4_ present in TG (and can also provide some T_3_) [68]. Residues orthologous to this tyrosine of human TG are conserved in other vertebrate species, where they serve as the primary site of T_4_ formation [88]. A tyrosine 125 residues downstream from this acceptor serves as the outer ring donor for thyroxine formation at this site, as dehydroalanine has been directly visualized in the cryo‐EM electron density map of native, iodinated bovine thyroglobulin [37]. Whereas an alternate tyrosine residue has been hypothesized to serve as another DIT donor to the same site [46], this seems unlikely because not only is dehydroalanine not found at the purported “alternate donor site” as observed for the donor 125 residues downstream from the Y24 acceptor, but the postulated “alternate donor” did not reveal iodinated tyrosine in the electron density map [46]. Rather, it appears that this “alternate tyrosine” serves as a key part of a binding pocket for thyroxine at Y24 [37].

Two additional conserved hormonogenic sites reside within the C‐terminal 200 residues of TG (“Sites B and C”) [68]. Acceptor Site B (Tyr2554) is also conserved [38, 89, 90] and is linked to a DIT donor 33 residues upstream [37]. Acceptor Site C within the short unique tail sequence at the extreme C‐terminus of TG (Tyr2747) is also conserved [38, 89, 90], and is thought to be a site preferential for T_3_‐formation [87, 91]. Iodination at this site can form both MIT (which makes it a possible donor residue in coupling) and DIT (a possible acceptor) [90, 92]. The ChEL domain [68] is critical to TG dimerization, and the short unique tail sequence that follows cannot be visualized in the cryo‐EM electron density map (and thus its precise positioning can only be inferred in Figure 5), consistent with peptide motion that may bring residue 2747 of one monomer into proximity with the same residue of the second monomer within the TG dimer. Thus, current thinking is that T_3_ formation within the short unique tail sequence involves an intermolecular coupling reaction between the monomers [87]. Indeed, T_3_ can be formed at this site even upon iodination of a dimeric secreted form of truncated TG bearing only the ChEL domain and its short unique tail sequence [87]. Finally, Tyr1291 and Tyr685 have also been posited as minor hormone‐forming sites [38, 46, 68, 89, 92] that might be used to diversify hormonogenesis in special circumstances (such as in Graves' disease)—but these and other tyrosine residues can also contribute to non‐hormonogenic iodide storage [68].

## 5 Thyroid Gene Defects and Dyshormonogenesis

Congenital hypothyroidism is the most common endocrine disease in childhood, with a prevalence of 1 in 2000–3000 live births [93]. Hypothyroidism caused by genetic mutations can be classified into two groups: (i) disorders of thyroid gland development (agenesis or dysgenesis), and (ii) disorders caused by defects in any of the steps of thyroid hormone synthesis (dyshormonogenesis), which generally account for up to 20% of cases [93]. As noted below, genetic defects affecting thyroid hormone synthesis can affect any of the four major steps: (i) iodide availability, (ii) hydrogen peroxide generation, (iii) oxidized iodide formation, and (iv) TG itself.

Mutations in *SLC5A5* (which encodes NIS) [94], *SLC26A4* (which encodes PDS) [95] and *SLC26A7* (which encodes another iodide transporter) [96] all affect iodide uptake into thyrocytes or iodide efflux to the thyroid follicle lumen. Mutations in *IYD* prevent normal intrathyroidal iodide recycling, leading to excessive thyroidal loss of MIT and DIT (with enhanced urinary excretion) and subsequent iodide deficiency [97].

Mutations in *DUOX2* or its associated maturation factor *DUOXA2* lead to insufficient hydrogen peroxide generation [98]. Interestingly, mutations in *DUOX1* or *DUOXA1* have not been linked to congenital hypothyroidism, again pointing out the small contribution of DUOX1 to thyroidal hydrogen peroxide generation in comparison to that of DUOX2 [97].

Mutations in *TPO* decrease the formation of oxidized iodide and thereby impair the iodination of tyrosyl residues in TG—this appears to be the most frequent cause of inherited dyshormonogenesis with permanent congenital hypothyroidism [99, 100, 101]. Cases of permanent hypothyroidism caused by *TPO* genetic defects are usually associated with goiter, with hypothyroidism inherited in an autosomal recessive manner [102, 103, 104]. Severe biallelic mutations in *TPO* lead to a total iodide organification defect, whereas heterozygous *TPO* mutants may exhibit a partial iodide organification defect [102, 105]. However, there are some *TPO* heterozygous patients with an apparent total iodide organification defect, suggesting the existence of *TPO* allelic interactions that may create more complex phenotypes [102, 105, 106, 107].

Mutations in *TG* are another commonly reported cause of congenital hypothyroidism, with at least 227 different human *TG* gene mutations identified to date [58, 108, 109]. Structurally defective (i.e., misfolded) TG mutants become entrapped in the ER, which impairs the protein trafficking to the follicle lumen that is necessary for normal thyroid hormone synthesis [110] (discussed further, below).

## 6 Effects of Primary Hypothyroidism on the Thyroid Gland

A decrease in adequate thyroid hormone synthesis and release triggers a rise in TSH that leads to several sequelae in the thyroid gland. TSH action on its receptor stimulates TG endocytosis from the apical membrane of thyrocytes, as well as stimulating the expression and activities of other gene products involved in thyroid hormone formation [68]. TSH binds to its G protein‐coupled receptor TSHR on the basolateral membrane of thyrocytes and stimulates cAMP synthesis via the guanine nucleotide‐binding protein G_αs_ [111]. Activation of the cAMP cascade stimulates the expression of NIS, TPO, and TSHR [112, 113]. In addition, TSH affects the intracellular trafficking of NIS [114], pendrin [115], TPO [116] and TG in thyrocytes [68] as well as DUOX association with TPO [116], mostly via protein kinase A (PKA)‐dependent pathways [115]. Additionally, TPO activity is enhanced [116] via increased production of H_2_O_2_ from the DUOX system through the G_αq_‐ G_α11_‐mediated phospholipase C (PLC)‐diacylglycerol‐calcium pathway [117]. Interestingly, hypothyroidism can stimulate a shift of hormone production from T_4_ to relatively more T_3_ by a combination of TSH effects on thyroidal iodination [54, 118] as well as increased enzymatic peripheral conversion of T_4_ to T_3_ [119, 120].

## 7 Homozygous and Heterozygous Mutant Thyroglobulin in the Thyroid Gland

As noted above, the mechanism of congenital hypothyroidism in the setting of biallelic *TG* mutation in both humans and murine models involves misfolding of TG, leading to its inability to undergo proper forward trafficking from the ER, resulting in failure to be secreted to the follicle lumen [110]. In the setting of biallelic *TG* mutation, feedback to the intact HPT axis results in chronic elevation of TSH [97], which drives thyrocyte proliferation [121] that can lead to goiter development as is seen both in human patients [122, 123, 124] and large animal models [125, 126]. Such behavior is also observed in *cog*/*cog* mice encoding TG‐L2263P [127] and *rdw*/*rdw* mice with a knock‐in in the mouse *Tg* locus encoding TG‐G2298R [128]. However, adult *rdw*/*rdw* rats (encoding the same TG‐G2298R missense mutation but in Wistar‐Imamichi rats that are derived from the WIC genetic background) [129] develop an overall thyroid gland that becomes hypotrophic or atrophic [130, 131] due to an inability to maintain thyrocyte proliferation in adulthood [128]. This was initially confused for secondary hypothyroidism, as low levels of growth hormone and prolactin were found in the pituitary of *rdw*/*rdw* rats, which was thought to be associated with a primary pituitary defect such as occurs with defects in the Pit‐1 transcription factor. However, studies eventually established that the observed pituitary effects are merely secondary consequences of severe hypothyroidism and do not reflect any defect in Pit‐1 mRNA or protein [132]. Rather, it has been found that some substrains derived from the WIC rat background are predisposed to defects in thyrocyte proliferation including thyroid dysgenesis [133]. Interestingly, some patients bearing biallelic *TG* mutations also present without goiter [134], strongly suggesting heterogeneity of the thyroid proliferative response in patients with different bi‐allelic *TG* mutations as well as different genetic backgrounds. In the setting of *rdw*/*rdw* rats, the inability of adult animals to generate sufficient thyroid cell proliferation to grow a large goiter correlates directly with the inability of these same animals to sustain circulating T_4_ levels [128]. In humans, there has been much consideration of the question of whether goiter development is a physiological adaptation [135] or maladaptation [136], but it would appear that in the setting of biallelic *TG* mutation [128], the ability to sufficiently and chronically sustain thyrocyte cell proliferation to develop a goiter may be required for generating and sustaining sufficient circulating T_4_.

**FIGURE 6:**
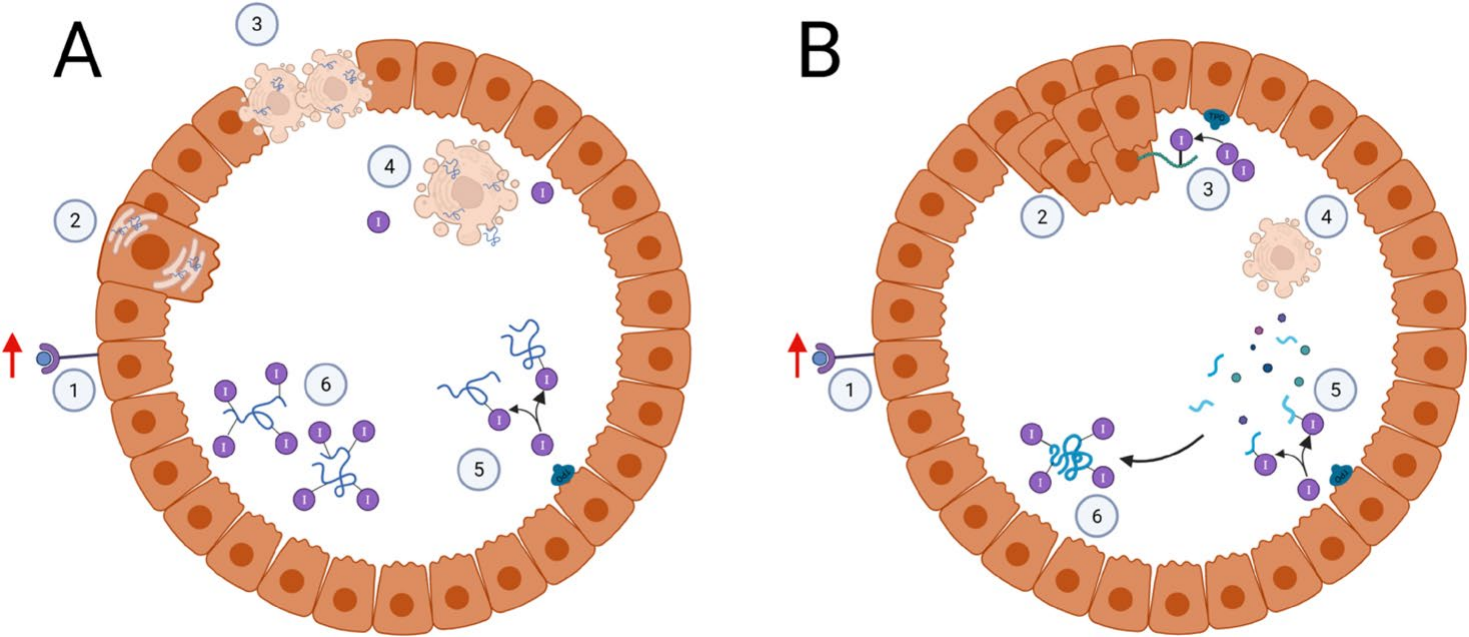
Two proposed mechanisms of thyrocyte cell death and thyroid hormone formation in patients expressing mutant thyroglobulin. (A) ER‐stress dependent model: Hyperstimulation by TSH (1) leads to increased expression of misfolded TG, causing chronic unremitting ER stress (2) followed by cell death (3). Dead thyrocytes then detach from the epithelial monolayer to enter the thyroid follicle lumen, releasing their contents (including mutant TG) (4) that can be iodinated by TPO (5) to form thyroid hormone (6). (B) Iodination‐mediated cell death model: Hyperstimulation by TSH (1) leads to overgrowth of thyrocytes into the follicle lumen (2). Iodination of the exposed surfaces of overgrown thyrocytes (3) is cytotoxic (4), releasing the contents of dead thyrocytes for iodination (5) and hormone formation (6). In both models, dead cell debris, including iodoproteins, are ultimately endocytosed and lysosomally digested by surrounding living thyrocytes, liberating T_4_ for transport to the bloodstream.

Along with thyrocyte proliferation that is required for goiter development, another notable hallmark of this disease is ongoing thyroid cell death. In congenital hypothyroidism with bi‐allelic *TG* mutation, dead thyrocytes are often found within the luminal cavity, and these cells can show activation of caspase‐3, cleavage of PARP, and positivity by TUNEL staining *en route* to complete nuclear fragmentation/karyolysis [110]. This cell death is chronic (ongoing throughout the course of the untreated disease), heterogeneous within follicles, and asynchronous, resulting in some dead cells inside the follicle lumen at various stages of decay and disintegration, yet surrounded by other living and dividing cells that can still participate in the follicular architecture that encloses iodoproteins within the apical cavity [110]. Interestingly, the complete disintegration of dead thyrocytes in the follicle lumen leads to the eventual exposure of all of the cell's constituents to the iodination environment [110]. Thus in untreated patients (or animals) with bi‐allelic mutant *TG*, dying and dead thyrocytes provide a supply of protein substrate that becomes available within the follicle lumen for iodination and, potentially, for the synthesis of T_4_ (Figure 6) [110].

Given this inefficient salvage mechanism, the blood level of circulating T_4_ in the untreated disease relies (indirectly) on total thyroid cell mass to provide sufficient cellular substrate (dead cells) for the synthesis of T_4_, such that increasing amounts of circulating T_4_ correlate with postnatal expansion of the thyroid gland [110]. These observations support the argument in favor of TSH‐dependent goiter development [135] as a key physiological adaptation in the untreated disease. Indeed, in *cog*/*cog* mice, massive postnatal expansion of the thyroid gland parallels a rise in serum T_4_ [137], which originates from hormonogenesis derived from the ghosts of dead thyrocytes [110]. Conversely, in *rdw*/*rdw* rats, it is the inability to maintain sufficient thyrocyte proliferation to develop a goiter [128] that corresponds to chronic profound hypothyroidism in untreated animals, because the hypotrophic thyroid gland simply cannot provide sufficient dead cells [110] to maintain the needed thyroid hormone production.

For those untreated patients (or animals) with bi‐allelic *TG* mutation that do develop a large goiter and are therefore able to generate significant circulating thyroid hormone levels [138], it is still unknown what protein(s) are used as the substrate for thyroidal T_4_ production. It is known that denatured TG is incompetent for thyroid hormone synthesis [139, 140]; thus thyroxine formation on misfolded TG entrapped in the ER is expected to be very low. Indeed, only very tiny amounts of T_4_ can be synthesized within the mutant Tg protein that is delivered to the follicle lumen via release from dead thyrocytes [110], and it is far from clear that this is sufficient to generate normal or near‐normal circulating T_4_ levels in goitrous patients. Although serum albumin has long been known to become iodinated in such patients [141]—delivered to the follicle lumen either by paracellular leakage [141] or by transcytosis [142]—iodination of albumin also generates vanishingly small amounts of T_4_ [143, 144]. Indeed, the recent recognition that the entire proteome of dead thyrocytes can become available within the follicle lumen creates new possibilities for potential protein substrate(s) that may be iodinated for T_4_ biogenesis in the natural history of this disease (considered further, below).

In patients with homozygous expression of mutant TG, high levels of ER stress have been observed with massive ER swelling that occupies the largest volume fraction within the cytoplasm and drives overall cell swelling—accompanied by a dramatic increase in ER stress markers including ER chaperones and oxidoreductases [128]. Classically, *TG* mutations are thought of as recessive, suggesting that there are no pathological phenotypes in patients expressing one wildtype *TG* allele [145]. These heterozygous patients may present with slightly elevated TSH but normal T_4_ levels, consistent with subclinical hypothyroidism, as is also seen in a mouse model bearing one WT *Tg* allele and one *cog Tg* allele [146]. The prevalence in the human population of heterozygous expression of pathogenic variants of *TG* is estimated to be quite common [147, 148], although these patients may never be diagnosed and may never even be symptomatic. Almost certainly, the overwhelming majority of T_4_ synthesized in these heterozygous patients (or animal models) derives from the wildtype TG protein that is successfully secreted to the thyroid follicle lumen. Nevertheless, despite the normal circulating T_4_ levels, it has now been observed that in mouse models, monoallelic mutant *Tg* expression also results in significant ER stress and thyroid cell death [146]. Indeed, even the living thyrocytes in such animals exhibit significant histological changes (ER and cell swelling and a diminished size of the thyroid follicle lumen) that can be largely rescued in animals treated with exogenous T_4_ to lower thyroid stimulation by TSH [146].

## 8 Thyroid Hormone Synthesis Before the Evolution of the Thyroglobulin Gene

Before evolving the ability to synthesize thyroid hormone, some organisms are thought to have obtained thyroid hormones from food sources and used the ingested thyroid hormones to regulate various physiological functions [149]. This hypothesis is supported by evidence that many common marine food sources such as algae, sponges, and corals, contain very large amounts of iodinated organic compounds [150]. In the case of sponges and corals, these compounds predominantly come in the form of T_4_ [150]. Thus, animals most likely evolved mechanisms to respond to these hormones even before the evolution of a specialized organ for endogenous T_4_ (and T_3_) synthesis [151, 152]. Thereafter, many phyla found ways to endogenously synthesize iodotyrosines before the evolutionary appearance of *TG* [150]. Indeed, the expression of iodotyrosine deiodinase (to recycle iodide from iodotyrosine) has been identified from mammals to bacteria [151]—while, as noted below, the expression of orthologs of thyroid peroxidase to support iodination activity is found in platyhelminths, ascidians, amphioxus, mollusks, echinoderms, and other animal lineages [153, 154, 155, 156].

Several modern invertebrates are capable (even if not efficient) of synthesizing thyroid hormone in the absence of expression of *TG* [150]. An example occurs in the amphioxus endostyle [157], an iodination‐competent pharyngeal organ [158] that is embryologically related to the vertebrate thyroid gland [159, 160, 161]. The amphioxus endostyle expresses thyroid‐related transcription factors known to serve as regulators of thyroid development such as Nkx2.1, FoxE4, and Pax8 [158, 162]. Additionally, the endostyle expresses several thyroid‐related proteins involved in iodine metabolism [158] and can generate proteinaceous T_4_ [162], the formation of which is blocked by methimazole—the same peroxidase inhibitor used to inhibit thyroid hormone synthesis in human patients [160]. Similarly, ascidian urochordates do not express a *TG* gene [161] yet synthesize T_4_ within the endostyle [163, 164]. Here too, other elements of the thyroid hormone system are present including orthologs of thyroid peroxidase [154, 165] as well as iodothyronine deiodinase [166]. Biosynthesis of thyroid hormone is not a coincidence but is actually central to the biology of both amphioxus and ascidians, in which thyroid hormone action controls metamorphosis [163, 167, 168]—whereas inhibition of thyroid hormone synthesis causes developmental defects and delays metamorphosis in a manner that can be rescued by administration of exogenous thyroid hormone [163, 169, 170]. Echinoderms are a phylum even more distant from chordates yet here too, T_4_ has been confirmed to exist [156, 171, 172]—and accelerates development and metamorphosis in sea urchins [156, 172, 173], sand dollars [3, 5, 171, 174], and starfish [175]. Interestingly, while some sea urchins obtain thyroid hormone exogenously from algal food sources [172], others have been found to synthesize thyroid hormone endogenously [5, 171]. Although no specific tissue sites have thus far been identified [150], thyroid hormone levels rise during larval life in echinoderms, peaking before metamorphosis [5]—and inhibition of thyroid hormone synthesis in sand dollars delays or prevents metamorphosis [5, 171]. Finally, thyroid hormone synthesis has been detected even in some protostomes such as sea hares [156], scallops [176], and oysters [177], and this is once again associated with development (gastrulation) and metamorphosis [177]. Altogether, it is clear that endogenous thyroid hormone synthesis, linked to organismal growth and development, can occur without specific expression of the *TG* gene.

## 9 Evolution of Thyroglobulin

Various domains of the TG protein are more ancient than full‐length TG [178, 179, 180]. Proteins containing TG‐type 1 repeats [180], and those bearing TG‐type 2 repeats have been found in amphioxus and sea urchin, but the identification of any of these proteins as potential TG orthologs has not been demonstrated [38]. In addition, the alpha‐beta hydrolase fold family (that includes the TG‐ChEL domain) is present in a variety of ancient proteins [179] but these have also not been established as TG orthologs. However, once the vertebrate phylum begins, TG becomes a highly conserved protein, suggesting a strong selection pressure to retain it [38].

Notably, the *Tg* gene is found even in the primitive vertebrate lamprey which diverged from other vertebrates > 500 million years ago [38, 181]. Amazingly, in the pharynx of lamprey larvae, there is exocrine secretion of one or more iodoproteins containing thyroid hormones through the endostyle [182], and during metamorphosis the endostyle becomes reorganized into a true endocrine thyroid gland that expresses *Tg* [182, 183, 184, 185]. It remains unclear whether TG protein expression first begins in the larval endostyle or only after the thyroid gland develops in mature lamprey [185], but the encoded hormonogenic tyrosines and the same overall sequence features and domain composition are all found in lamprey *Tg* [38, 88] with many additional tyrosines available for iodide storage. It is notable that while ancestral lampreys lived in the sea where iodide is abundant, lampreys migrated and evolved to live in fresh water (with an iodide concentration that is at least an order of magnitude lower than seawater). Thus, the simplest interpretation is that thyroid follicular storage of TG began and co‐evolved with the need for iodide storage in the body once animals were exposed to an environment with decreased iodide availability [38]. One cannot exclude that fully functional and efficient TG‐like thyroid hormone‐ and iodide‐storage proteins already pre‐existed [186]—if so, one or more ‘partial *Tg*’ genes should be identifiable in invertebrates—but so far this has not been found [38].

## 10 Hormone Synthesis in the Thyroid Gland That Lacks Secretable Thyroglobulin Is Built on Several Biological Processes Known to Occur in Other Body Tissues

Interestingly, in untreated goitrous patients with homozygous *TG* defects [138], as well as goitrous mice bearing biallelic *Tg* mutations, serum thyroid hormone levels can be surprisingly normal or near‐normal [128]. The emerging explanation for hormonogenesis in this setting involves massive cellular proliferation and goiter growth driven by high circulating TSH, accompanied by cell death of thyrocytes released into the follicle lumen—with subsequent iodination of the dead cell “ghosts” to produce thyroid hormone for secretion to the rest of the body [128]. This may be viewed as a productive form of cell death akin to holocrine secretion—a mode that involves the release of the entire contents of dead cells as observed in the exocrine glands of reptiles, birds, and mammals [187]. While cell death is often thought of as an adverse consequence, it is a required mechanism that serves many important functions in all multicellular organisms [188] and generally occurs by apoptosis [189]. First, we offer several examples in which holocrine secretion is vitally important in the human body, providing evidence that dead cells may benefit the organism.

In skin sebaceous glands, sebocytes [190, 191, 192, 193] form a sac‐like, multilayered epithelium connected to hair follicles [194]. Cells in the basal layer proliferate while the inner suprabasal cells activate a programmed cell death pathway for release of sebum into the hair canal [194]. Dead sebocytes undergo autolysis driven by released lysosomal enzymes, and release of DNAse helps to degrade the dead‐cell DNA, accompanied by the complete disintegration of sebocytes [194]. A related type of cell death has been reported during mammary gland involution after lactation [195]. Further, there are species such as the Amazonian river prawn in which androgenic hormone secretion requires cell death and degeneration [196]. Additionally, in the meibomian glands of the eyelid—basal acinar cells differentiate and move toward the apical center of the acinus before holocrine secretion [197]. The harderian gland provides a similar secretory function in the orbit [198, 199, 200] and, in animal studies, oxidative stress can trigger the holocrine secretion [201]. Moreover, in the human uterus after ovulation [202]—although not a classical example—recent electron microscopic observations indicate that endometrial epithelial cells exhibit features of apocrine and possibly holocrine secretion [203, 204], which is hypothesized to play a role in facilitating embryo implantation [204]. Indeed, recent studies have described that this spontaneous cell death is reduced in the endometrium of unexplained‐infertile women [205].

Altogether, cell death can serve as an important part of an organism's flexibility in maintaining homeostasis [206] even as it can appear in various disease states. Certainly, apoptosis can be triggered by DNA damage [207], oxidative stress [208], and mitochondrial dysfunction [206], and in the thyroid has been linked to conditions including thyroid cancer [209], Hashimoto's thyroiditis [210, 211], and Graves' disease [212]. Thyrocytes are also susceptible to non‐apoptotic forms of cell death [213]. Necrosis is a form of cell death structural breakdown and leakage of cellular contents into the surrounding tissues leading to inflammation [206] that has been reported in various types of thyroid cancers [214, 215]; while pyroptosis (a form of inflammatory programmed cell death) has been found to be associated with thyroiditis and autoimmune thyroid disorders [216]. Importantly, in epithelial cells including thyrocytes, loss of close basolateral plasma membrane contact with the extracellular matrix predisposes to a form of cell death referred to as anoikis [217], a process to which normal thyrocytes are susceptible [whereas thyroid cancers develop resistance] [218].

Patients bearing biallelic *TG* mutations (as well as animal models—even including those with monoallelic disease) exhibit abundant thyrocyte cell death [110, 128, 146]. The cause of cell death in this pathogenic condition has not been clearly established, but it has long been assumed to be due to toxic levels of ER stress. Under this proposed mechanism, mutations in *TG* lead to misfolding of the protein [219] that is monitored through ER stress sensors such as IRE1, PERK, and ATF6, which transmit responses leading to decreased global translation while the expression of ER chaperones such as BiP and other protein folding factors is increased [219], accompanied by ER stress‐associated autophagic degradation of misfolded proteins [220]. In the setting of irremediable ER stress, cell death pathways can become activated [221, 222].

As an alternative, thyrocyte overgrowth into the follicle lumen may predispose thyrocytes to both anoikis as well as cytotoxicity from enhanced exposure to the luminal environment—such factors must also be considered as potential contributors to cell death. As noted above, hydrogen peroxide is abundant in the thyroid follicle lumen, although thyrocytes ordinarily have defenses such as host enzymes to resist its cytotoxicity [26]. However, the thyroid follicle lumen also presents a specialized environment rich in oxidized iodide (for which degradative host enzymes are not known to exist) and which may be present at inordinately high levels in the setting of *TG* mutation, because TG protein is no longer secreted into the follicle lumen. To state more explicitly, in normal individuals, secreted TG protein in mass quantity competes very successfully for oxidized iodide, thereby “absorbing” most of the iodination [223]. In the absence of secreted TG in the follicle lumen, the increased availability of free oxidized iodide beyond that in normal thyroid follicles [224] favors renegade iodination of other proteins and lipids. Before discussing renegade (non‐TG) iodination as a potential trigger of thyroid cell death, let us briefly consider two other examples of peroxidase‐catalyzed cytotoxicity that set a precedent in the body.

Some monocytes/macrophages and neutrophils express myeloperoxidase (MPO), a heme peroxidase [225, 226, 227, 228, 229]. After phagocytosis [230], these myeloid cells generate hydrogen peroxide [226, 231, 232] and then use MPO for both peroxidation and ortho‐halogenation to generate toxic oxidants [233, 234] that kill bacteria and other invading pathogens [235]. Specifically, during the ortho‐halogenation cycle, MPO can oxidize chloride (Cl^−^), bromide (Br^−^), and thiocyanate (SCN^−^) to generate hypochlorous acid (HClO), hypobromous acid, and hypothiocyanite, respectively [233, 234, 236, 237]. MPO preferentially oxidizes Cl^−^ because of its higher concentration in the bloodstream [236] and the resulting HClO can modify lipids, DNA, polysaccharides, glycosaminoglycans, and amines including tyrosine [238], leading to fragmentation of each of these classes of macromolecule [239, 240, 241, 242, 243, 244, 245, 246].

In another example, the luminal epithelial cells of the lactating mammary gland express lactoperoxidase (LPO), a heme peroxidase associated with antibacterial and antifungal activity [247, 248, 249]. Like thyroid peroxidase and myeloperoxidase, LPO utilizes hydrogen peroxide as an electron acceptor. LPO forms hypothiocyanite (OSCN^−^) [250, 251] and can oxidize I^−^ as well as Br^−^ (LPO does not use Cl^−^) [252, 253]. LPO‐catalyzed iodination tends to target thiol/sulfhydryl groups of peptides and proteins, as well as NAD(P)H, thereby inhibiting bacterial metabolism [250].

Finally, we return to mouse models lacking thyroglobulin (*Tg‐KO*), in which abundant thyrocyte cell death is observed, in a TSH‐dependent manner [224]. In this setting, the levels of ER stress markers have actually been found to be lower than that observed in the thyroid gland of normal wildtype animals [224], so cell death due to increased ER stress is highly unlikely. In the complete absence of TG, the animals exhibit very low circulating T_4_ levels in early postnatal life, with extraordinary TSH elevation [254]. However, after the mice grow to adulthood, concomitant with massive goiter growth, these animals (like patients or animals with bi‐allelic *TG* defect) eventually normalize their circulating T_4_ levels [254]. In the absence of TG protein, T_4_‐containing protein is synthesized with low efficiency on the ghosts of dead thyrocytes (with T_3_ generated from circulating T_4_ deiodination) [254]. Notably, the thyroidal content of T_4_‐containing protein is not appreciably different in the presence of homozygous mutant TG or in the complete absence of TG [254]. Whereas immunoblotting with T_4_‐specific antibodies demonstrates T_4_‐containing protein in normal thyroid to be exclusively TG and its proteolytic fragments, in thyroid glands of *Tg‐KO* or *rdw*/*rdw* or *cog*/*cog* mice (the latter bearing homozygous mutant TG protein), such immunoblotting shows a similar pattern, revealing only a weak broad smear of low‐efficiency T_4_‐containing proteins [254]. Thus, the profile of a multitude of low‐efficiency T_4_‐containing protein bands (which is blocked in animals treated with the iodination inhibitor PTU) appears similar in thyroid tissues of mice bearing mutant TG or no TG at all. Each of these mutant strains of mice exhibit on the order of ~1% efficiency in generating T_4_‐containing protein per unit thyroid tissue (which does not improve throughout life), but remarkably, the total yield of thyroidal T_4_ output slowly improves with sufficient goiter growth [254].

Based on these findings, the simplest hypothesis is that in patients with bi‐allelic thyroglobulin mutation, TSH‐driven iodination can support inefficient T_4_ formation derived from the iodoproteome of dead thyrocytes. We posit that such that untreated individuals can exhibit substantial circulating T_4_ provided that they have developed a goiter sufficient to generate an ample supply of dead cells needed for this inefficient hormonogenesis mechanism. Interestingly, inhibition of iodination—whether through limiting *Tg‐KO* animals' iodide consumption, treating with propylthiouracil (PTU) to inhibit thyroid peroxidase, or by genetic knockout of the H_2_O_2_‐generating DUOX system—all were found to inhibit thyroid cell death (Figure 6B) [224]. Moreover, analogous to the halogenation mechanisms described above, direct in vitro exposure to iodide plus an H_2_O_2_‐generating system was found to directly kill thyrocytes in culture, but only when peroxidase activity was present—indicating that renegade iodination itself can trigger thyrocyte cell death in a manner that does not involve ER stress [224]—and such iodination does weakly generate T_4_‐containing protein [254]. Nevertheless, most patients with biallelic or monoallelic *TG* mutations do have substantial thyroidal ER stress, suggesting that iodination‐mediated cell death and ER stress‐mediated cell death may co‐exist in the thyroid glands of patients (and animals) expressing misfolded mutant TG.

## 11 Conclusion

Thyroid hormone is important for growth, development, and the maintenance of homeostasis in many organisms including humans. In all vertebrates, TG synthesized and secreted by thyrocytes is the major precursor protein used for thyroid hormone synthesis. However, it is possible to synthesize thyroxine without TG, as has been demonstrated in invertebrates that do not express the *Tg* gene but evidently express other hormonogenic gene products that can serve as iodination substrates for which the degree of efficiency in thyroid hormonogenesis has not been established. What is clear, however, is that in vertebrates, because of its high intrinsic capability for thyroid hormone synthesis as well as iodide storage, TG has been evolutionarily selected for its ability to undergo secretion in order to become iodinated in the thyroid follicle lumen.

Patients bearing biallelic *TG* mutations cannot secrete the protein to the follicle lumen. Remarkably, untreated goitrous patients, as well as animal models, can synthesize sufficient thyroid hormone levels to sustain life—some even achieving normal or near‐normal circulating thyroxine levels. While serum albumin had previously been hypothesized to serve as the back‐up protein substrate for thyroid hormone synthesis in such patients or animal models, more recent findings point to a different explanation that involves thyroid cell death. Dead thyrocytes released to the follicle lumen disintegrate and become iodinated, leading to eventual exposure of all thyrocyte proteins within the iodination environment. Such a mechanism is inherently inefficient as most iodoproteins have very limited hormonogenic potential, and for this reason, success in thyroid hormonogenesis in the absence of secretable TG depends upon a large supply of dead cells to the lumen of thyroid follicles, which in turn depends upon the growth and maintenance of a thyroid goiter needed to generate the cellular mass to sustain such delivery.

In humans, thyrocyte cell death has already been shown to occur in many thyroid pathologies. However, there are many different kinds of cell death that are likely to be linked to different thyroid disease states. In patients with biallelic *TG* mutations, thyrocyte death has generally been attributed to ER stress [110]. Although ER stress has certainly been associated with cytotoxicity in various diseases, other cell death drivers may contribute to the phenotype of dead thyrocytes in the thyroid glands of patients with biallelic *TG* mutations. In particular in this disease, dead thyrocytes are found in the thyroid follicle lumen detached from their basement membrane and contained within a uniquely cytotoxic environment. Whereas the apical membrane of polarized thyrocytes is expected to protect cells from the cytotoxic environment of the follicle lumen, TSH‐stimulated overgrowth of thyrocytes into the lumen risks anoikis and increased cell exposure to the iodination environment, both of which may contribute to cell death. It has still not been determined the extent to which, in patients bearing *TG* mutations, thyrocytes first die from ER stress and then are shed into the follicle lumen, or if they first are lost to the follicle lumen and then become increasingly damaged by the cytotoxic environment, where they die. Either scenario in patients with biallelic *TG* mutations ultimately positions dead thyrocytes in follicle lumen where the ghosts of dead thyrocytes become available as substrate for iodination and are competent (albeit inefficiently) to support thyroid hormone synthesis. Mutant TG undoubtedly contributes something to this inefficient hormone production, but other inefficient hormonogenic substrate proteins derived from dead thyrocytes are likely to provide the bulk of the hormone synthesis, essentially driving the TSH‐stimulated thyroid goiter of these patients toward a holocrine secretion mechanism.

## Author Contributions

Writing the initial manuscript (C.Y.); supervision (P.A.); editing the manuscript and approving the final draft (both).

## Funding

This work is the result of NIH funding and is subject to the NIH Public Access Policy. Through acceptance of this federal funding, the NIH has been given a right to make the work publicly available in PubMed Central. This work was supported by NIH R01DK132017 (to P.A.) and F30DK139717 (to C.Y.).

## Conflicts of Interest

The authors declare no conflicts of interest.

## Data Availability

The authors have nothing to report.
